# Supplementary material for: Protein Sectors: Statistical Coupling Analysis versus Conservation
Source: PLoS Comput Biol. 2015 Feb 27;11(2):e1004091. doi: 10.1371/journal.pcbi.1004091 (PMC4344308; doi:10.1371/journal.pcbi.1004091)
Supplement: S1 Text — (PDF) [file pcbi.1004091.s004.pdf]

Protein sectors: statistical coupling analysis versus conservation.
Supporting information

Tiberiu Teşileanu, Lucy J. Colwell, and Stanislas Leibler

Contents

1 Calculating sequence covariance **1**

2 Statistical coupling analysis **5**

3 Alignments **6**

4 Details about the DHFR analysis **8**

5 Alanine scans **8**

6 Diagonal of SCA matrix instead of conservation **9**

7 Multiple sectors **9**

8 Top eigenmode of SCA matrix—some details **13**

1 Calculating sequence covariance

Let us assume we are given a multiple sequence alignment of homologous proteins as an $N \times n$ matrix A_{ki}. In order to calculate correlations in the alignment, we need a way of transforming it to numeric data. There are three main ways in which this was done in the literature [1–5], which we describe below; generally all these methods yield highly similar results. In the present work, we used the projection method (item 3 below), which is the default in the latest SCA release from the Ranganathan lab. Note that SCA requires positional weighting to be done on top of the covariance analysis. This is described in the next section.

1. **The binary approximation.**

   We start by describing the simplest approach, which constructs a "binary approximation" of the alignment [1, 2]. In the binary approximation, each amino acid is replaced by 1 if it is equal to the consensus amino acid at its position, and 0 otherwise,

   $$X_{ki} = \delta(A_{ki}, c_i)\,, \tag{S1}$$

   where the consensus amino acid c_i is the most frequent amino acid found in column i of the alignment.[1] Here A_{ki} is the amino acid found at position i in sequence k, and the Kronecker symbol $\delta(a, b)$ is 1 if and only if $a = b$.

   The covariance matrix is then defined in the standard way,

   $$C^{\text{bin}}_{ij} = f_{ij} - f_i f_j\,, \tag{S2}$$

   where

   $$f_{ij} = \frac{1}{N}\sum_k X_{ki}X_{kj}\,, \qquad f_i = \frac{1}{N}\sum_k X_{ki}\,, \tag{S3}$$

   with N being the number of sequences in the alignment. Note that f_i is simply the frequency of the consensus amino acid at position i, which is often used as a measure of the conservation level at that position. Also, f_{ij} is the frequency at which the consensus amino acids occur simultaneously in the two columns i and j.

2. **The reduction method.**

   The most generic statistical analysis that can be performed with categorical data is using *contingency tables*. In this context, these are tables of the frequencies at which various combinations of amino acids occur simultaneously in a sequence—we can, for example, define the frequency $f_i(a)$ with which amino acid a is found at position i, and the frequency $f_{ij}(a, b)$ with which amino acids a and b co-occur at positions i and j, respectively. It is then convenient to define a "binary representation" of the alignment [5], $x_{ki}(a)$, where $x_{ki}(a)$ is equal to 1 if A_{ki} is a, and 0 otherwise; in short,

   $$x_{ki}(a) = \delta(A_{ki}, a)\,. \tag{S4}$$

   Note that here there are 21 columns for each column in the original alignment. It is important to keep in mind that—despite the potentially confusing nomenclature—the binary representation $x_{ki}(a)$ is very different from the binary approximation X_{ki}. The former is an exact representation of the alignment data, while the latter is an approximation that keeps only part of the information in A_{ki}.

[1] This is typically restricted to non-gaps, though in practice this usually does not affect the results.

The single-site and pairwise frequencies, $f_i(a)$ and $f_{ij}(a,b)$, are thus averages involving the binary representation

$$\begin{aligned} f_i(a) &= \frac{1}{N}\sum_k x_{ki}(a)\,, \\ f_{ij}(a,b) &= \frac{1}{N}\sum_k x_{ki}(a)x_{kj}(b)\,. \end{aligned} \tag{S5}$$

Now we can define the covariance

$$C_{ij}(a,b) = f_{ij}(a,b) - f_i(a)f_j(b)\,. \tag{S6}$$

For each pair of sites, we obtain a covariance value between every pair of amino acids, a and b. There are 21 choices of amino acid (including the gap), so for each pair of sites (i,j), C_{ij} is a 21×21 matrix. Note, however, that frequencies are normalized—$\sum_a f_i(a) = 1$ and $\sum_b f_{ij}(a,b) = f_i(a)$—which means that not all numbers in the covariance matrix are independent. In fact, if we are given the values of $C_{ij}(a,b)$ for all amino acids except one (commonly, the gap), it is straightforward to infer the missing values. For this reason, unless otherwise stated, we will always assume a and b to exclude the gap, and will treat the matrix C_{ij} as having size 20×20 instead of 21×21.

In the binary approximation, for each pair of sites i and j, we were able to calculate one number C_{ij}^{bin} showing the amount of covariance between the sites. In contrast, the full covariance matrix $C_{ij}(a,b)$ contains an entire matrix of numbers for each pair of sites. It is sometimes useful to collapse this matrix to a single number that measures the overall covariance between two sites, as was the case in the binary approximation. This is usually done using a heuristic approach, for example by using the Frobenius norm [6]

$$C_{ij}^{\mathrm{red}} = \left[\sum_{a,b} C_{ij}^2(a,b)\right]^{1/2}\,, \tag{S7}$$

or the spectral norm [3]

$$C_{ij}^{\mathrm{red}} = \text{largest singular value of } C_{ij}(a,b)\,. \tag{S8}$$

The binary approximation described above can actually also be seen as a reduction method, in which

$$C_{ij}^{\mathrm{red}} = C_{ij}(c_i, c_j)\,, \tag{S9}$$

with c_i being the consensus amino acid in column i, as before.

Another approach for carrying out the reduction starts from a ratio of frequencies,

$$D_{ij}(a,b) = \log\frac{f_{ij}(a,b)}{f_i(a)f_j(b)} = \log\left[1 + \frac{C_{ij}(a,b)}{f_i(a)f_j(b)}\right]. \tag{S10}$$

The mutual information is then a natural measure of the independence of two random variables that can be constructed from D [7,8],

$$\mathrm{MI}_{ij} = \sum_{\substack{a,b\\ \text{incl. gaps}}} f_{ij}(a,b) D_{ij}(a,b) = \sum_{\substack{a,b\\ \text{incl. gaps}}} f_{ij}(a,b)\log\frac{f_{ij}(a,b)}{f_i(a)f_j(b)}. \tag{S11}$$

Note that here we include gaps in the summation because the definition of the mutual information given in eqs. (S10) and (S11) assumes that $f_i(a)$ and $f_{ij}(a,b)$ are proper frequencies; in particular, they should sum up to 1, and this is only true if the values for the gaps are included in the sum.

3. **The projection method.**

Instead of calculating the full covariance matrix and then reducing it, the projection method starts by projecting the binary representation onto an $N \times n$ numeric matrix Y_{ki},

$$Y_{ki} := \sum_a x_{ki}(a) v_i(a), \tag{S12}$$

where v_i are unit vectors. We can then use the definitions (S2) and (S3) with Y instead of X to obtain the covariance matrix,

$$C^{\text{proj}}_{ij} = \frac{1}{N}\sum_k Y_{ki}Y_{kj} - \frac{1}{N^2}\sum_{k,l} Y_{ki}Y_{lj}. \tag{S13}$$

The projection vectors that we use in this paper are given by [4]:

$$v_i(a) = \frac{f_i(a)}{\sqrt{\sum_b f_i(b)^2}}. \tag{S14}$$

Note that, as we did above for the covariance matrix, here too we do not include the gap, *i.e.,* we assume that $v_i(\text{gap}) = 0$, and we have $b \neq \text{gap}$ in the summation.

It is interesting to note that a different choice for the v_i can be used to recover the binary approximation,

$$v_i^{\text{binary}}(a) = \delta\big(a, \arg\max_{b\neq\text{gap}} f_i(b)\big) \equiv \delta(a, c_i). \tag{S15}$$

For both the binary approximation and the projection method, the approximation is most accurate for highly conserved sites.

2 Statistical coupling analysis

There are two main features that distinguish SCA from a standard covariance analysis: the introduction of positional weights and the positivity of the matrix elements. Starting again with the binary approximation, for simplicity, the covariance matrix C defined in (S2) is transformed to [1]

$$\tilde{C}_{ij} = |\phi_i \phi_j C_{ij}| \, , \tag{S16}$$

where the weights ϕ_i are specific functions of the single-site statistics, chosen based on the idea that entries of the covariance matrix corresponding to poorly conserved sites are less likely to be informative than those corresponding to highly conserved sites, and thus should be given less weight.

The absolute value that appears in the formula was justified by the requirement of finding blocks of coevolving residues regardless of the sign of the correlations [1]. It also avoids a certain instability to small perturbations that appears in cases in which the consensus amino acid has a frequency that is very similar to that of the next most common amino acid. In these cases, a small perturbation in the alignment can flip the order of the top amino acids, thus flipping the sign of some correlations in the binary approximation.[2] Taking the absolute value may, however, introduce artifacts, as described in the main text.

The expression commonly used for the positional weights is [1, 2, 4]

$$\phi_i = \log \left[\frac{f_i}{1 - f_i} \frac{1 - q(c_i)}{q(c_i)} \right] \, , \tag{S17}$$

where f_i is the conservation at site i, *i.e.* the frequency in the alignment of the consensus amino acid, while $q(c_i)$ are background frequencies for the consensus amino acids c_i. Note that we are assuming that the background frequencies depend only on the identity of the consensus amino acid, and not on the position i in the protein. These background expectations can be estimated by averaging over a large set of proteins. The functional form (S17) for the positional weights was chosen to match the original 1999 formulation of SCA [1, 9] and to fulfill the role of down-weighting poorly conserved sites, but is otherwise arbitrary.

Instead of performing the positional weighting at the level of the covariance matrix, it could have been performed on the binary alignment itself. The covariance matrix of the transformed binary alignment

$$\tilde{X}_{ki} = \phi_i X_{ki} \tag{S18}$$

directly yields $\tilde{C}_{ij}$, after taking the absolute value of each element. This can be generalized to apply to the binary representation matrix; we define

$$\tilde{x}_{ki}(a) = \phi_i(a) x_{ki}(a) \, , \tag{S19}$$

[2] We thank O. Rivoire for this observation.

in which we replace (S17) by [5]

$$\phi_i(a) = \log\left[\frac{f_i(a)}{1-f_i(a)}\frac{1-q(a)}{q(a)}\right] . \tag{S20}$$

With this positionally-weighted binary representation we can use either the reduction method [5] or the projection method [4] described in the previous section. When using the projection method, the absolute value of each element is taken when calculating the SCA matrix, as is done with the binary approximation. An interesting empirical observation is that the mutual information defined in eq. (S11) is well-approximated by a weighted SCA matrix using the reduction method, in which the positional weights are chosen equal to the logarithm of the frequencies.

For the results in the main text, after the SCA matrix was calculated, the sector positions were identified by placing a threshold on the components of its top eigenvector. Note that, due to the positivity of the elements of the SCA matrix, the Perron-Frobenius theorem guarantees that all these components are positive.

3 Alignments

As described in the Methods section of the paper, the software package `HHblits` [10] was used to generate the alignments used in the the main text. We also analyzed a number of alignments from other sources to make sure the results weren't sensitive to this choice. For the cases of PDZ [4], DHFR [3], and the potassium channels [11], we ran the analysis on the alignments that were used in the papers that first applied the SCA method to those proteins. For PDZ and DHFR we also ran the analysis on Pfam alignments (`PF00595` and `PF00186`, respectively). This was not done for the potassium channels and *lacI* because no suitable alignments were available in Pfam. See Table 1 for a summary of the results.

To improve the quality of the alignments, it has been suggested [5] to filter them by removing repeated sequences, sequences with too many gaps, and positions with too many gaps. From our tests, however, these procedures do not make a big difference to the results of this paper. For this reason, the only filtering we perform is to remove the insert states from `HHblits` alignments, which in our case amounts to removing columns containing 40% or more gaps.

In addition, for the PDZ alignment from McLaughlin Jr. et al. [4], we removed the columns containing more than 20% gaps, because this is how that alignment was processed in the scripts provided by the authors of that article [4]. There is a minor glitch in the procedure of mapping alignment columns to PDB coordinates in McLaughlin Jr. et al. that leads to the misidentification of one of the columns (corresponding to PDB position 334). For consistency with the older work, we worked with this minor error in the alignment, but we checked that the results are not significantly affected by it.

Table 1: Results of the analysis performed in this paper when run on different alignments.

Alignment	Contingency table for sector[a]			Contingency table for conservation			Comparison (χ^2 p value)
		**S**	**NS**		**C**	**NC**	
PDZ [4]	**F**	15	5	**F**	15	5	$p_{\chi^2} = 1.00$
	**NF**	8	53	**NF**	8	53	
		**S**	**NS**		**C**	**NC**	
PDZ (Pfam)	**F**	13	7	**F**	9	11	$p_{\chi^2} = 0.45$
	**NF**	7	52	**NF**	11	48	
		**S**	**NS**		**C**	**NC**	
DHFR[b] [3]	**F**	14	0	**F**	12	2	$p_{\chi^2} = 0.30$
	**NF**	33	17	**NF**	27	23	
		**S**	**NS**		**C**	**NC**	
DHFR[c] (Pfam)	**F**	14	0	**F**	12	2	$p_{\chi^2} = 0.29$
	**NF**	35	15	**NF**	29	21	
		**S**	**NS**		**C**	**NC**	
potassium channels[d] [11]	**F**	18	19	**F**	18	19	$p_{\chi^2} = 0.96$
	**NF**	17	67	**NF**	20	64	

[a]**C**, **S**, and **F** stand for conserved, sector, and functional, respectively.

[b]For DHFR we are counting the residues that are "touched" by either sector or conserved residues. Although the number of conserved residues we are considering is equal to the number of residues in the sector, the number of surface residues that are "touched" is different.

[c]See footnote *b*.

[d]The number of residues in the SCA sector is the same as the number of conserved residues we considered. The sum of the entries in the **S** column does not match that in the **C** column because some of the sector residues are located at sites where we have no experimental data.

4 Details about the DHFR analysis

The way in which the DHFR alignment was analyzed by Reynolds et al. [3] has a number of peculiarities compared to the other datasets we presented, which we describe below. We note, however, that their results are not significantly different from those obtained with our simplified protocol.

The SCA method used by Reynolds et al. was the spectral-norm reduction method described above (see section 1, item 2) using a thresholded form of the positional weights [3],

$$\phi_i(a) = \begin{cases} \log\left[\dfrac{f_i(a)}{1 - f_i(a)} \dfrac{1 - q_a}{q_a}\right] & \text{for } f_i(a) > q_a, \\ 0 & \text{else.} \end{cases} \tag{S21}$$

Furthermore, the SCA matrix was "cleaned" by subtracting the average SCA matrix calculated for 100 randomized alignments. Each of the randomized alignments was obtained by independently permuting the elements of the alignment columns, which has the effect of destroying correlations without affecting the single-site amino acid frequencies.[3]

The sector was defined by the residues for which the component of at least one of the top five eigenvectors goes above a given threshold [3]. To select the threshold, first a Student's t-distribution was fit to the components of each of the eigenvectors, and then the value for which the t-distribution PDF drops below a certain threshold was used as a cutoff. The PDF threshold is given by p_i for the ith eigenvector, where p_i is[4]

$$p_i = \frac{0.005}{2\,\mathrm{IQR}(v_i) n^{-1/3}}\,, \tag{S22}$$

where $\mathrm{IQR}(v)$ is the interquartile range of v (the range over which the middle half of the components of v spread), and v_i is the ith eigenvector. Despite the complicated selection procedure, a very similar sector can be obtained by using a constant PDF threshold for each of the top five eigenvectors, or even just by using the largest components of only the top eigenvector.

5 Alanine scans

Instead of using all the experimental data for PDZ and for *lacI*, we can restrict our attention to alanine mutations, to get an idea for the amount of information contained in an alanine

[3]Since the number of random samples is finite, the results depend slightly on the state of the random number generator. The results from the original paper by Reynolds et al. can be obtained by using the default random number generator in Matlab with the default seed.

[4]The dependence of the threshold on i is an artifact of the fact that Reynolds et al. applied a constant threshold to histogram values instead of PDF values, and the bin size for the histograms was determined using the Freedman-Diaconis rule [12], and thus varied between eigenvectors. This can be seen from the Matlab scripts provided by the authors.

Table 2: The results from the paper when restricting to alanine mutations.

Alignment	Contingency table for sector			Contingency table for conservation			Comparison (χ^2 p value)
		**S**	**NS**		**C**	**NC**	
PDZ	**F**	10	10	**F**	9	11	$p_{\chi^2} = 0.98$
	**NF**	11	50	**NF**	12	49	
		**S**	**NS**		**C**	**NC**	
lacI	**F**	16	26	**F**	20	22	$p_{\chi^2} = 0.82$
	**NF**	65	221	**NF**	61	225	

scan. As mentioned in the paper, the qualitative results do not change much, though, as expected, the quality of the match between the predictions from SCA or conservation and the experimental data is reduced (see Table 2).

6 Diagonal of SCA matrix instead of conservation

In the paper we point out that the top eigenvector of the SCA matrix correlates primarily with the diagonal elements $\tilde{C}_{ii}$ of this matrix (or rather, with their square root), while the correlation with conservation is weaker. This is because, although both conservation and the diagonal elements of the SCA matrix can be calculated from the single-site frequencies $f_i(a)$, the relation between them is non-trivial and non-monotonic. We therefore wondered how the functional significance of the sector residues compared to that of residues that have high values of $\tilde{C}_{ii}$. The results can be found in Table 3.

7 Multiple sectors

There are two key questions related to multiple SCA sectors: one is how to determine how many eigenvectors to analyze, and the second one is which linear combinations of eigenvectors to use for finding sectors. We briefly discussed the second question in the main text, and pointed out that while there are several approaches that have been used in the literature to find the appropriate linear combinations, these approaches have little or no theoretical motivation and have been tested only in a very limited fashion.

In the main text, we pointed out that we can instead use linear regression to find the

Table 3: The results of the paper when replacing conservation by the diagonal of the SCA matrix.

Alignment	Contingency table using $\tilde{C}_{ii}$			Comparison to sector (χ^2 test p value)
		**C**	**NC**	
PDZ	**F**	12	8	$p_{\chi^2} = 0.87$
	**NF**	9	52	
		**C**	**NC**	
DHFR	**F**	13	1	$p_{\chi^2} = 0.61$
	**NF**	34	16	
		**C**	**NC**	
potassium channels	**F**	16	21	$p_{\chi^2} = 0.95$
	**NF**	16	74	
		**C**	**NC**	
lacI	**F**	43	27	$p_{\chi^2} = 0.55$
	**NF**	38	220	

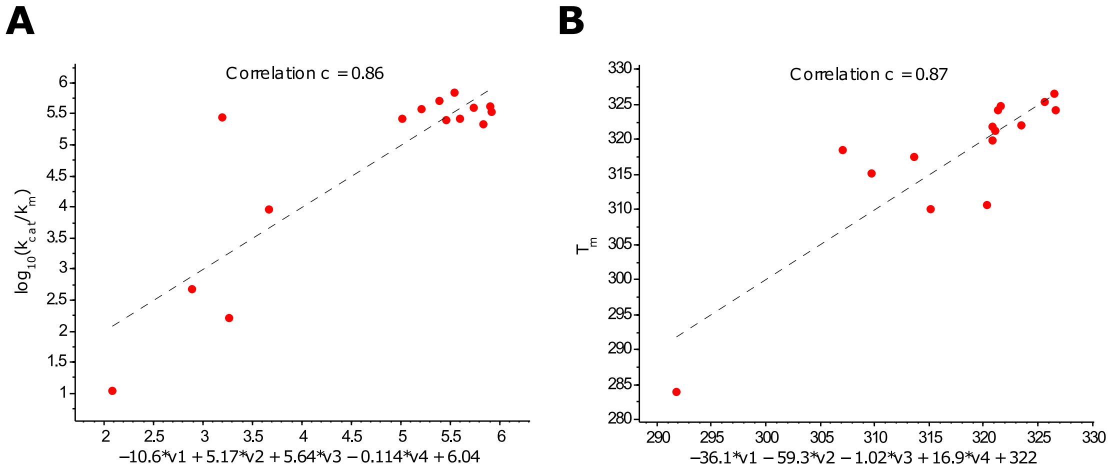

Figure S1: **SCA top eigenvectors fit to trypsin data.** We attempt to fit **A.** binding affinity, or **B.** denaturation temperature for the single mutants of rat trypsin described in Halabi et al. [1] against the components of the top four eigenvectors of the SCA matrix corresponding to the mutated residues. The best linear regressions are shown on the x-axis. The dashed line has slope 1 and intercept 0.

linear combinations that best approximate the measured quantities.[5] Here we show the results of such an analysis for the case of serine protease (Figure S1), PDZ (Figure S2), and the potassium channels (Figure S3). While for serine protease the regression works well for both measured quantities, for PDZ we can only fit the mutational effect on binding to the CRIPT ligand, and for potassium channels the fit is not very good to either the activation potential V_{50} or the equivalent charge z.

The number of eigenvectors to consider for sector determination is itself a difficult problem. It was addressed by Halabi et al. using an approach inspired from the analysis of financial markets [1]. Essentially, the eigenvalue spectrum of the SCA matrices obtained for randomized alignments was obtained, and a threshold was established at the upper edge of this distribution. Any eigenvalues of the SCA matrix for the real alignment that fell below this edge were considered noise and were ignored. This approach was motivated by the Marčenko-Pastur distribution, that describes the eigenvalue spectrum for a covariance matrix $C = X^T X$ associated to a random i.i.d. data matrix X. However, the assumption of identically distributed elements does not hold for alignment data, because different columns in the alignment have different amino acid distributions, even when there are no correlations between columns. For this reason, the spectrum of the SCA matrix does not resemble the

[5] This of course assumes that the relation is linear, which is far from obvious, but can be thought of as a first-order approximation.

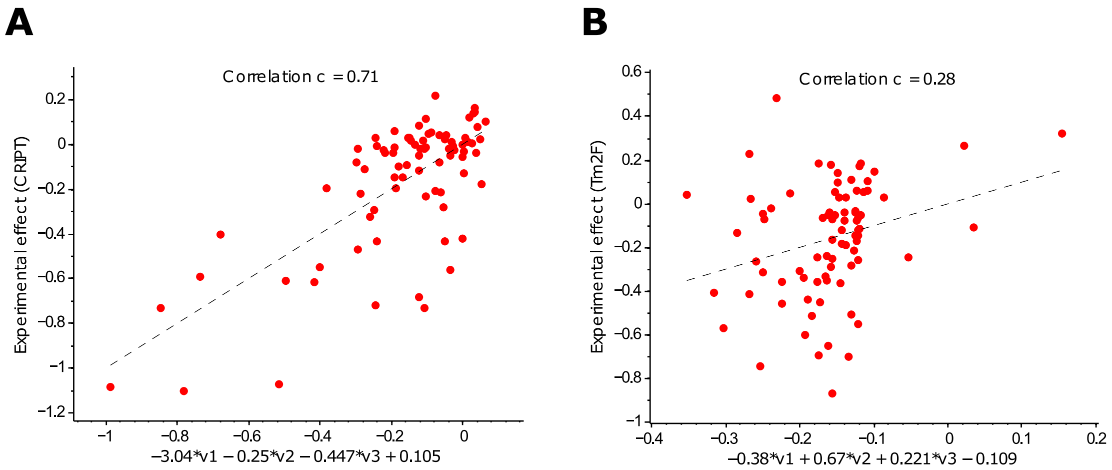

Figure S2: **SCA top eigenvectors fit to PDZ data.** We attempt to fit the measured mutational effect for binding to **A.** the CRIPT ligand, or **B.** the $T_{-2}F$ ligand as measured for the single mutants of $PSD95^{pdz3}$ described in McLaughlin Jr. et al. [4] against the components of the top three eigenvectors of the SCA matrix corresponding to the mutated residues. The best linear regressions are shown on the x-axis. The dashed line has slope 1 and intercept 0.

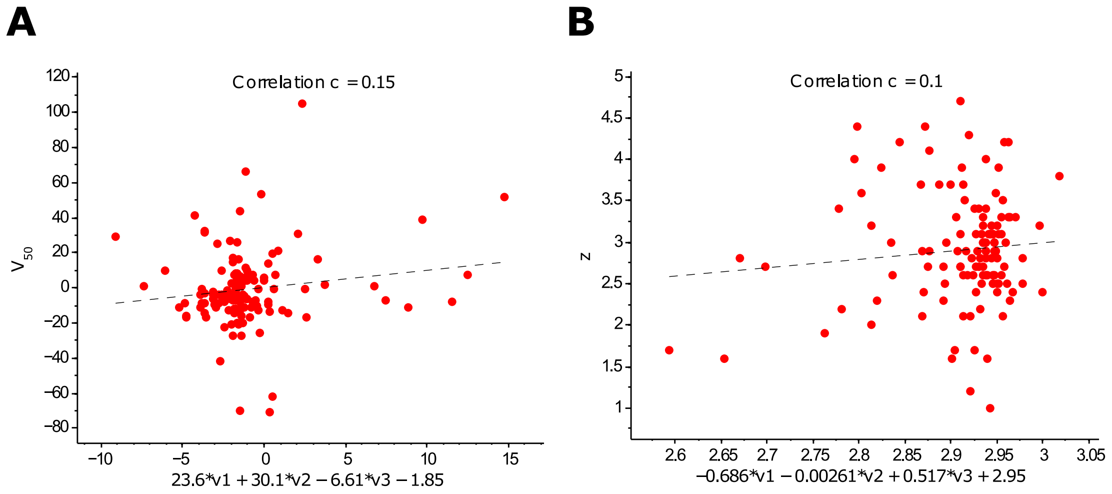

Figure S3: **SCA top eigenvectors fit to potassium channels data.** We attempt to fit **A.** the activation voltage V_{50}, or **B.** the equivalent charge z measured for single mutants of the *drk1* voltage-gated K^+ channel in Li-Smerin et al. [13] against the components of the top three eigenvectors of the SCA matrix corresponding to the mutated residues. The best linear regressions are shown on the x-axis. The dashed line has slope 1 and intercept 0.

Marčenko-Pastur distribution, and intuitions based on this distribution need not hold in the case of SCA. In particular, eigenvalues that are below the edge obtained from randomized samples could contain perfectly valid information about the protein.

Another difficulty with SCA is that the absolute value of all the elements in the covariance matrix is taken, which makes the top eigenvector become an outlier. As described in the main text, although this top eigenmode is much over the "noise" edge described above, the information it contains is essentially independent of correlations, and is thus indistinguishable from noise.[6]

8 Top eigenmode of SCA matrix—some details

Here we fill in some of the details for the model presented in the paper that can explain the correlation between the components of the top eigenvector of the SCA matrix and its diagonal entries. As in the paper, suppose we have a covariance matrix with off-diagonal

[6]This is because the randomized alignments used with SCA, which are used to calculate the noise distribution, are obtained by shuffling elements in the alignment columns. This keeps the single-site frequencies intact while destroying correlations, implying that the single-site frequencies are a feature of the noise model.

entries that are biased towards positive values. The simple model we wrote for this is

$$M = \begin{pmatrix} d_1^2(1+x) & d_1 d_2 x & \cdots & d_1 d_n x \\ d_2 d_1 x & d_2^2(1+x) & \cdots & d_2 d_n x \\ \cdots & \cdots & \ddots & \cdots \\ d_n d_1 x & d_n d_2 x & \cdots & d_n^2(1+x) \end{pmatrix} \equiv \begin{pmatrix} \Delta_1 & d_1 d_2 x & \cdots & d_1 d_n x \\ d_2 d_1 x & \Delta_2 & \cdots & d_2 d_n x \\ \cdots & \cdots & \ddots & \cdots \\ d_n d_1 x & d_n d_2 x & \cdots & \Delta_n \end{pmatrix} . \tag{S23}$$

For simplicity, let us assume that there are no degeneracies between the d_i, *i.e.,* that $d_i \neq d_j$ for $i \neq j$, and that M is not singular, *i.e.,* $d_i \neq 0$ for all i. Let $v = (v_1, \ldots, v_n)$ be an eigenvector of this matrix with eigenvalue λ. Then we have

$$d_i^2 v_i + d_i x \sum_j d_j v_j \stackrel{!}{=} \lambda v_i \,, \tag{S24}$$

which yields[7]

$$v_i = \Big(\sum_j d_j v_j \Big) \frac{d_i x}{\lambda - d_i^2} \,. \tag{S25}$$

This implies that the components of the eigenvectors are related to the diagonal elements $\Delta_i = d_i^2(1+x)$ by

$$\frac{\sqrt{\Delta_i}}{v_i} \propto \lambda - \frac{\Delta_i}{1+x} \,. \tag{S26}$$

If we multiply eq. (S25) by d_i and sum over i, we can divide through by $\sum_j d_j v_j$, and get

$$1 = x \sum_i \frac{d_i^2}{\lambda - d_i^2} \,, \tag{S27}$$

which can be used to estimate λ. In particular, this equation allows us to show that between each consecutive pair of values d_{i_1} and d_{i_2}, there is exactly one eigenvalue.

By the Perron-Frobenius theorem, the top eigenvector can be chosen to have all components positive, and thus it should have λ larger than all d_i^2. Assuming $\lambda \gg d_i^2$, which empirically seems to be the case for SCA matrices, we get an estimate for the top eigenvalue

$$\lambda_{\text{top}} \approx x \sum_i d_i^2 = \frac{x}{1+x} \sum_i \Delta_i \equiv \frac{x}{1+x} \operatorname{Tr} M \,. \tag{S28}$$

[7]We may worry about division by zero. Note that, from the eigenvalue equation (S24), $\lambda = d_i^2$ for some i if and only if $\sum_j d_j v_j = 0$ (since we assumed all $d_i \neq 0$). However, feeding this back into eq. (S24), we see that this is only possible if all the v_j for which $d_j^2 \neq \lambda$ are zero. Since we assumed that none of the d_j vanish, $\sum_j d_j v_j = 0$ can only hold if at least two components of v are non-vanishing. This, however, would imply that there is a degeneracy, which we explicitly disallowed. We thus conclude that $\lambda \neq d_i^2$ for any i.

It should be checked that this is consistent with the condition that λ is much larger than all d_i^2; this seems to be true for empirical SCA matrices. The Perron-Frobenius theorem also guarantees that all other eigenvectors of M will have both positive and negative components, and therefore, according to eq. (S25), the corresponding eigenvalues will have to be smaller than the largest d_i^2. This implies that for the SCA matrices, the top eigenvector will be an outlier, *i.e.,* the SCA matrices are approximately rank-1, which can indeed be observed for real alignments.

Using $\lambda_{\text{top}} \gg d_i^2$ in eq. (S25), we get

$$v_{i,\text{top}} \approx \Big(\sum_j d_j v_{j,\text{top}}\Big)\frac{x}{\lambda_{\text{top}}} \times \sqrt{\Delta_i}\,, \tag{S29}$$

which is the observed linear relation between the top eigenvector and the square root of the diagonal elements of the SCA matrix. This argument shows that the top eigenvector is strongly correlated with single-site statistics and thus largely independent of correlations between positions. It is important to emphasize that this does not mean that there is no information contained in this mode, but only that most of this information can be obtained without any analysis of correlations.

As mentioned in the paper, we emphasize again that in this derivation the origin of the off-diagonal entries is not specified. They could be an artifact of sampling noise, they could come from actual non-specific correlations between positions, or they could be due to a non-trivial phylogenetic structure of the alignment, as suggested by Halabi et al. [1].

References

[1] Halabi N, Rivoire O, Leibler S, Ranganathan R (2009) Protein sectors: evolutionary units of three-dimensional structure. Cell 138: 774–786.

[2] Smock RG, Rivoire O, Russ WP, Swain JF, Leibler S, et al. (2010) An interdomain sector mediating allostery in Hsp70 molecular chaperones. Molecular Systems Biology 6: 414.

[3] Reynolds KA, McLaughlin Jr RN, Ranganathan R (2011) Hot spots for allosteric regulation on protein surfaces. Cell 147: 1564–75.

[4] McLaughlin Jr RN, Poelwijk FJ, Raman A, Gosal WS, Ranganathan R (2012) The spatial architecture of protein function and adaptation. Nature 491: 138–142.

[5] Rivoire O (2013) Elements of Coevolution in Biological Sequences. Physical Review Letters 110: 178102.

[6] Ranganathan R, Rivoire O (2012). Note 109: A summary of SCA calculations. Available online at `http://systems.swmed.edu/rr\_lab/Note109\_files/Note109\_v3.pdf`.

[7] Fodor AA, Aldrich RW (2004) Influence of conservation on calculations of amino acid covariance in multiple sequence alignments. Proteins 56: 211–21.

[8] Dunn SD, Wahl LM, Gloor GB (2008) Mutual information without the influence of phylogeny or entropy dramatically improves residue contact prediction. Bioinformatics 24: 333–40.

[9] Lockless SW, Ranganathan R (1999) Evolutionarily Conserved Pathways of Energetic Connectivity in Protein Families. Science 286: 295–299.

[10] Remmert M, Biegert A, Hauser A, Söding J (2012) HHblits: lightning-fast iterative protein sequence searching by HMM-HMM alignment. Nature Methods 9: 173–175.

[11] Lee SY, Banerjee A, MacKinnon R (2009) Two separate interfaces between the voltage sensor and pore are required for the function of voltage-dependent K(+) channels. PLoS Biology 7: 676–686.

[12] Freedman D, Diaconis P (1981) On the histogram as a density estimator: L2 theory. Zeitschrift fuer Wahrscheinlichkeitstheorie und Verwandte Gebiete 57: 453–476.

[13] Li-Smerin Y, Hackos DH, Swartz KJ (2000) Alpha-helical structural elements within the voltage-sensing domains of a K(+) channel. The Journal of General Physiology 115: 33–49.
